# Molecular Method Based on Hydrolysis Probe Assays to Identify the Sex Chromosomes of Iberian Desman (*Galemys pyrenaicus*) Using Non‐Invasive Sampling

**DOI:** 10.1111/1749-4877.12933

**Published:** 2024-12-08

**Authors:** Adriana Ripa, María Jesús Palacios‐Gonzalez, José A. Díaz‐Caballero, Antonio Espinosa, Francisco Javier Zalba, Juan Luis García‐Zapata, José Luis Fernádez‐García

**Affiliations:** ^1^ Genetic and Animal Breeding, Faculty of Veterinary Universidad de Extremadura Cáceres Spain; ^2^ Dirección General de Sostenibilidad Junta de Extremadura Mérida Spain; ^3^ Área del Medio Natural, Sociedad de Gestión Pública de Extremadura (GPEX) Junta de Extremadura Mérida Spain; ^4^ Department of Mathematics Universidad de Extremadura Badajoz Spain

**Keywords:** DBX/DBY, *Galemys pyrenaicus*, non‐invasive samples, RT‐qPCR, sex identification

## Abstract

Desmans belong to the subfamily Desmaninae, which are members of the family Talpidae. Desmans and moles show limited sexual dimorphism, making unclear sex discrimination by phenotypic assessment. The Iberian desman (*Galemys pyrenaicus*) is an endangered species with a severe population decline. Knowledge of sex and sex ratio is essential for conservation and management. Based on these arguments and although previous conventional PCR studies amplifying DBX/DBY genes were relatively successful in sexing the desman, high‐resolution sex‐specific PCR has been requested. All these reasons encouraged us to develop new species‐specific RT‐qPCR assays by TaqMan probes to determine the sex in desman, especially with genetic material from non‐invasive samples. Accordingly, efficiency, limit of detection (LOD), specificity, and DNA analysis from faeces were verified. The target genes DBX and DBY were amplified with gDNA from both sexes, with Y‐chromosome consistently absent in the female. Despite the modest efficiency, regression analysis (*R* ^2^ > 0.999) indicated a linear range of the DBX and DBY assays extending from 20 to 0.2 ng/µL DNA. LOD analyses estimated that twice as much gDNA was needed in males as in females for DBX detection. Paradoxically, the Y‐chromosome required three times as much gDNA as the X‐chromosome using a male sample. Therefore, an unexpected dosage imbalance in the genome in favour of the X chromosome was discussed in light of an apparent multicopy nature of the DBX gene and with a sexing success rate of 49.9% of the non‐invasive samples, supporting Fisher's principle for the mammalian XX/XY sex system, as expected.

## Graphical abstract

To monitor populations of an endangered species such as *Galemys pyrenaicus* in the Extremadura area, qPCR techniques have been used to determine sex, using non‐invasive sampling techniques.

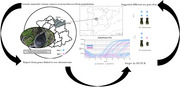

## 1 Introduction

Many taxa have accumulated substantial phenotypic differentiation between the sexes during evolution, except for the family Talpidae. Some of its species are among those that show unclear gender distinction, causing great controversy in sex determination by conspicuous anatomical dimorphism (González‐Esteban et al. 2003; Vidal et al. 2010). Desmans belong to the subfamily Desmaninae, which is part of the family Talpidae. Although molecular data have shown that the Eurasian mole and desman lineages diverged 37 million years ago (Douady and Douzery 2003), these species show little sexual phenotypic dimorphism confounding differentiation based on external anatomical assessment with the naked eye (González‐Esteban et al. 2003; Vidal et al. 2010). Previous studies on this issue reported inconsistencies and difficulties in determining undoubtedly the sex of the desman, especially in juvenile specimens (Peyre 1957a). In desmans, methods of sex identification have been described based on observation of oestrus in females and the presence of an open vaginal orifice with some perineal depigmentation (Richard and Micheau 1986) in this state. It is also possible to identify the sex of the male from the urinary papilla, which, when pressed lightly, shows a conical gland, while in females, the papilla appears long and narrow and has a notch at its terminal portion (González‐Esteban et al. 2003). Furthermore, an outstanding case of sex reversal has occurred in *Talpa occidentalis*, where XX individuals are functionally fertile females showing invariably ovotestes rather than normal ovaries (Jiménez, Burgos, and Barrionuevo et al. 2023), which may give reason to believe that the females could be true hermaphrodites. Along these lines, current research has highlighted a major challenge in linking genomic variation with phenotypic traits in evolutionary genetics (Real et al. 2020). However, reports are pointing out the additional inconveniences and concerns that come with live capture of threatened species, so the use of non‐invasive techniques aimed ultimately at identification through species‐specific genetic testing has been promoted (Ripa et al. 2023, 2024). However, individual sex assessments are among the most challenging genetic tests in those species that present little sexual dimorphism. For this reason, their relevance in the conservation of threatened species such as the desman has been considered undeniable (Allendorf et al. 2012 and references cited therein). Thus, early sex determination in endangered populations is of particular relevance when considering conservation programmes and population ecology studies. Sex identification techniques have proven useful in evolutionary biology, ecology, and conservation genetics studies of other species such as birds (Wang et al. 2010). Given the conservation status of our target species, it is vital to develop techniques to help us determine the sex of the desman for conservation planning purposes.

To address both shortcomings and errors associated with phenotypic methods, DNA‐based procedures have been developed, including non‐invasive molecular techniques. The advent of molecular genetic methods for desman sexing was pioneered by Vidal et al. (2010), who claimed high‐resolution and sex‐specific PCR techniques based on polymorphisms found in the DBX/DBY genes of the desman. These last authors suggested that their methods were less risky than those previously mentioned. Since then, the DBX/DBY genes have supported the sexing of desman by amplifying two DNA fragments involved in sex differentiation, which were found to be located on the X and Y chromosomes, respectively. At the same time, the same authors acknowledge the low efficiency of end‐point PCR in the sexing of this species when the source of genetic material was excreta. In this context, additional efforts are currently demanded to improve the deficiencies of the genetic tools previously used in sex determination in the desman (Vidal et al. 2010).

An important milestone in the use of PCR was the introduction of real‐time DNA amplification by monitoring the fluorescence produced in each cycle, now called real‐time PCR (RT‐PCR; Holland et al. 1991). In RT‐PCR, the point at which the fluorescence intensity increases above the detectable level is called the quantification cycle or threshold cycle (Cq), which allows the determination of the DNA concentration or the number of copies of the gene (Yang and Rothman 2004; Kubista et al. 2006; Bustín et al. 2009). At the same time, RT‐qPCR can also provide high specificity using probes‐based technologies (additional oligonucleotide: the probe) due to the lower susceptibility to visualize non‐specific PCR products (Bustin 2000; Kubista et al. 2006). Thanks to the advances that have been made in genetic techniques in recent years, it has been possible to specifically study the XX/XY sex‐determination system by RT‐qPCR. A sex determination test based on a single gene (e.g., Sry) could lead to the misidentification of males as females and result in PCR failures (Robertson and Gemmell 2006). This drawback has been partially solved by using two genes (e.g., DBX/DBY), instead of a single gene, for molecular sex determination tests. Even species‐specific designed primers have been combined in a multiplex PCR and tested on high‐quality DNA samples. In this way, the expected gene fragments for both males and females were amplified simultaneously (Vidal et al. 2010).

In summary, prior to the use of end‐point PCR described by Vidal et al. (2010), sex determination in this species was based on phenotype, although not without controversy as explained for more than five decades (Peyre 1956, 1957a; Richard and Micheau 1986; Palmeirim and Hoffman 1983; Nores et al. 1998; González‐Esteban et al. 2003). Later, Vidal et al. (2010) described the first sex‐determination protocol in the Iberian desman using end‐point PCR followed by sequencing using the Sanger method to identify genetic variation attributable to the sex chromosomes X (in males and females) and Y (only in males), but failure was also reported with faecal genetic material. All these reasons encouraged us to find new, efficient, sensitive and species‐specific RT‐qPCR methods for sexing desman and their useful application to detect sex chromosomes with DNA from non‐invasive samples.

## 2 Materials and Methods

### 2.1 Study Area

The study was carried out in the western part of the Central System of the Iberian Peninsula (Autonomous Community of Extremadura, Spain) in 2021. Faecal samples (*n* = 178) were collected according to their morphological characteristics by qualified staff in three geographically protected areas along the riverbeds in Ambroz Valley, Jerte Valley and La Vera (Tietar Valley). Sampling zones were delimited with four, eight and three sampling points, respectively, as described and established in the “Recovery Plan for the Desman” (*Galemys pyrenaicus*) in the Central System (Iberian Peninsula) in Extremadura (DOE 158 08/14/2018) (see Ripa et al. 2023, 2024). The sample collection sites cover sites of 300 m in length. Once established, within each area, the excrement collection points are proposed and separated from each other by approximately 100 m if necessary. All samples were geotagged, but by the decision of the regional government, the availability of the geolocation data is restricted due to the worrying state of the conservation status of the species in the sampling area.

### 2.2 Transport, Preservation, and Sex of Reference Samples

Faecal samples were transported in sterile tubes containing ethanol (96% or higher) and stored at −20°C during collection in the field and at −70°C upon arrival at the laboratory. Sex of reference DNA was isolated from tissues of a dead desman (male) from the La Vera population that was accidentally found in Extremadura (Spain) (Ripa et al. 2024) and a dead female from a conservation project (ADEFFA, personnel communication) preserved in 70% ethanol at −74°C.

### 2.3 Environmental DNA (eDNA) and Genomic DNA (gDNA) Isolation Assessing and Dilutions

Non‐invasive faecal eDNA was isolated using the QIAamp Fast DNA stool mini kit (QUIAGEN GmbH, Germany) according to the manufacturer's instructions (Ripa et al. 2023, 2024). Briefly, the contents of the collection tubes were centrifuged at 13,000 rcf/4°C, and the preservative ethanol was rejected with barrier‐tipped micropipettes in a dedicated UV cabinet. Additional manipulations of the total stool using the collection tube itself (2–5 mL in size) in the disinhibition step were avoided because the sample (faeces) weight typically ranged from 60 to 220 ng. However, a few large faeces were treated proportionally according to the manufacturer's recommendations (pg 30 of the protocol kit). Genetic material from each of the faecal samples was analysed to determine if they belonged to the desman following Ripa et al. (2024).

Genomic DNA (gDNA) was extracted from tissue (male and female desman) using a salting‐out DNA extraction procedure and diluted in molecular biology grade water, for the collection of quality genetic material as in Ripa et al. (2024).

DNA purity was assessed using a NanoDrop 2000 spectrophotometer (Thermo Fisher Scientific). Additionally, gDNA from tissues was further quantified using a Qubit 4 Fluorometer with dsDNA Assay Kit (Thermo Fisher Scientific) for both male and female DNA and, also, each subsequent decimal dilution series (up to the fluorometer sensitivity limit of 0.01 ng/µL) to adjust concentration dedicated to quantitative RT‐qPCR assays. The first template gDNA dilution was adjusted to 20 ng/µL, and then five additional serially 10‐fold dilutions were prepared (from 20 to 0.2 × 10^−3^ ng/µL) and concentration recalibrated as reported in this paragraph. Finally, the genetic material was stored at −70°C until use.

### 2.4 Hydrolysis Probes Design

The DBX and DBY gene sequences from Vidal et al. (2010) were used to design two primer pairs and two hydrolysis probes for RT‐qPCR assays using Primer3 software (amplicon size 148 and 120 for DBY and DBX, respectively; Table S1). Probes were designed containing a SQY quencher chemistry at the 3’ end and a 5' end reporter dye FAM or ABY for DBX and DBY gene marker, respectively (primers and probes available upon request), following manufacturer's recommendations (Thermo Fisher Scientific—Service & Support Solution; personal communication).

### 2.5 Primer/Probe Adjustment

Briefly, primers and probes were assayed using the basic reaction in Ripa et al. (2024). First, DBX and DBY assays (primers and probe) were mixed with 0.5 forward (F) +0.5 reverse (R)/ 0.6 probe (P) µL of 10 µM solution. Triplicate reactions were performed with 1 to 3 µL in a 10‐µL master mix containing 1 ng DNA of female and male gDNA. However, DBY showed more than 1 Cq higher than DBX from male gDNA. Then, DBY was tested with five condition concentrations using both excess primers or probes in triplicate. The mixture of 0.8 F + 0.8 R/0.7 P µL of 10 µM solution was selected. Finally, 1 µL of this assay was added to the basic reaction of a total of 10 µL, separately, to amplify the DBY and DBX genes as described below.

### 2.6 RT‐qPCR Assay

RT‐qPCR assays were performed on 96‐well plates with adhesive film or strips (Thermo Fisher Scientific, Waltham, MA) as required. Data were generated by RT‐qPCR using either a Step One Fast Plus or an ABI QuantoSudio3 real‐time PCR system thermal cycler (Applied Biosystems, Thermo Fisher Scientific) with standard curve acquisition using Step One software. But in the case of eDNA the presence/absence analysis was performed using Design and Analysis (DA2) Software ver 2.2.1 (Thermo Fisher Scientific Waltham, MA). A 10‐µL final reaction mixture was prepared containing 4 µL TaqMan TM Multiplex Master Mix (Applied Biosystems), 1 µL each of the adapted primer/probe assays and 5 µL DNA as template, whichever experiment (see Ripa et al. 2023, 2024). Each assay contained no‐template control (NTC) wells, including three or four replicates in each gene and a different template. All reactions for sex specificity and efficiency were performed in triplicate or quadruplicate. The amplification protocol used the following cycling programs Stage 1‐Hold with 50°C for 2 min and 95°C for 10 min and Stage 2‐PCR with fluorescence of 42 cycles of 95°C for 15 s, 62°C for 1 min, with fluorescence signal acquisition. The same cycling program was performed separately for each gene.

### 2.7 Efficiency, Limit of Detection (LOD), Specificity and Data Analysis in Faeces

**FIGURE 1:**
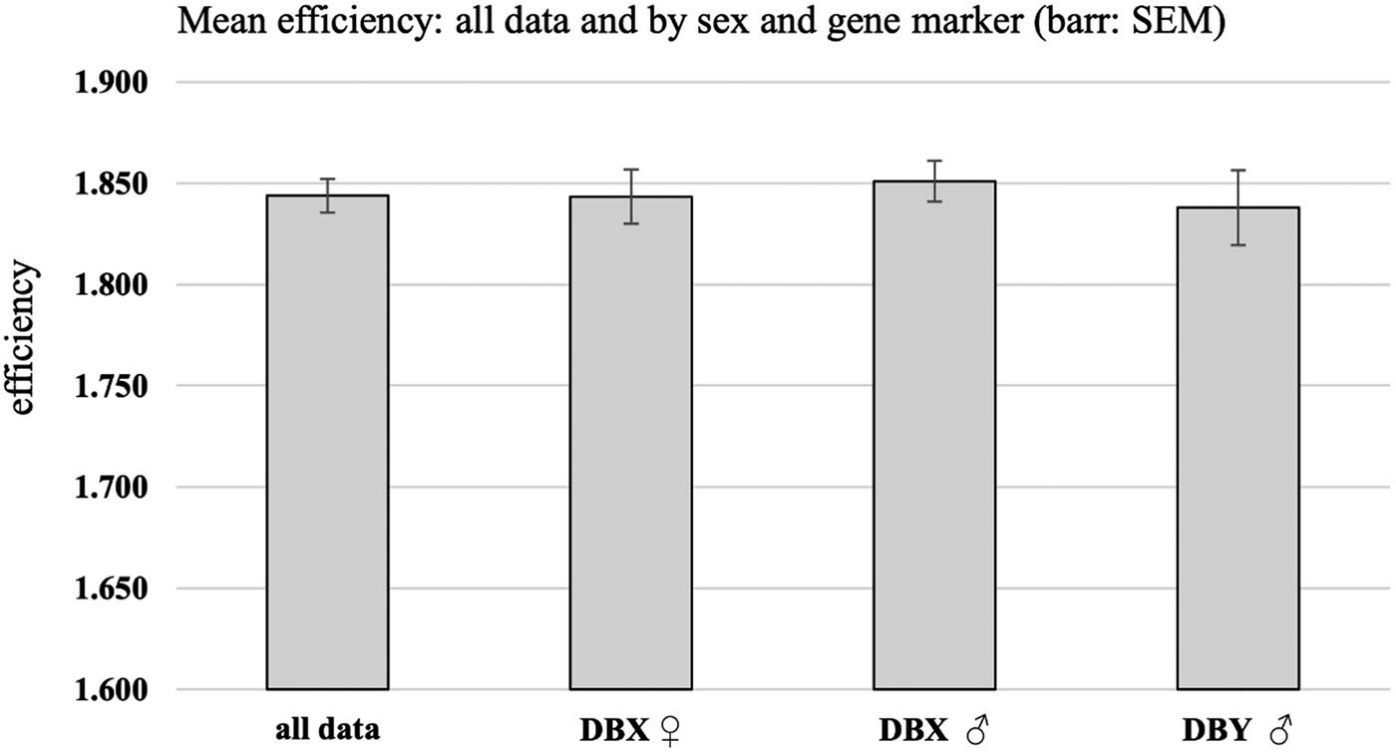
Mean efficiency based on 10‐fold dilution series of gDNA (three points) using all data sets and data by sex from DBX assays in females and in males and DBY assay in males.

The efficiency of the assays was checked following the methods of the LinRegPCR software ver. 2021.2 (https://www.medischebiologie.nl/files/; Ruijter et al. 2009) with template gDNA. This method calculates individual efficiency values for each qPCR reaction, and then, these are averaged globally and within every serial dilution for each target gene, both DBX assays (male and female) and DBY assays in the male desman to obtain average efficiency values (Figure 1 and Figure S1). Specifically, six decimal serial dilutions were adjusted from 100 to 10^−3^ total ng DNA/reaction (four technical replicates) with the aim of investigating the efficiency of the assay. The male and female gDNA was amplified for DBX and DBY separately using the same plate. Raw fluorescence signal results were exported from the ABI Quanto Sudio3 real‐time PCR system to the LinReg PCR‐based software as input data. The baseline of the fluorescence estimate was automatically determined and manually corrected, if necessary, for samples with low amplification signals. The W‐o‐L (Windows of Linearity) was then analysed to calculate individual and mean PCR efficiencies per group as follows: (1) global assays with all amplicon samples, all amplicons of DBX from male and female, that is, within individual sex and all DBY from male and (2) amplicons by every dilution group within sex and chromosome when male (Figure 1 and Figure S1). These input data for efficiency determination follow the recommendations of Ruijter et al. (2009). The last threshold cycle (Cq) was set to 40 in LinRegPCR software ver. 2021.2 to standardize a common threshold setting for all assays in the exponential phase of all reactions following Ruijter et al. (2009). Also, triplicate NTCs were used for each gene and sex.

The LOD of the assays was determined by preparing a series of 10 twofold dilutions extending the non‐linear range of the assay, which started from the 10^−1^ dilution (0.2 ng/µL) prepared earlier. Thus, each twofold dilution was prepared in triplicate and repeated three times each (nine repetitions total) for DBY and DBX both male and female gDNA. In this RT‐PCR, the presence/absence module was used with DA2 software according to its guidelines (Design & Analysis Software Ver. 2.6.0; Thermo Fisher Scientific). Statistical LOD analysis was performed using the Probit option in IBM SPSS Statistics ver. 27 under UEx License.

Presence/absence experiments were used to check the analytical specificity of each assay with reference female and male offspring and with different mammalian gDNAs (see Table 1) from our laboratory, including micromammal species inhabiting near the sampling territory such as *Neomys anomalus* and *T. occidentalis* (Ripa et al. 2024).

**TABLE 1:** Species used as controls, these results showed the tests performed on negative, positive (male desman), stencilled and unstencilled (NTC) specimens. The results were as expected, but the negative results on *Talpa occidentalis* and *N. anomalu*s are noteworthy because both species can be a real source of confusion.

Samples (Sex)	Cr X	Cr Y
*Oryctolagus cuniculus (♂)*	−	−
*Neovison vison*	−	−
*Canis lupus familiaris (♂)*	−	−
*Mus musculus (♂)*	−	−
*Lynx pardinus (♂)*	−	−
*Homo sapiens (♂)*	−	−
*Bos Taurus (♂)*	−	−
*Felis silvestris(♂)*	−	−
*Lutra lutra*	−	−
*Ovis orientalis aries (♂)*	−	−
*Talpa europaea*	−	−
*Neomys anomalus (♂)*	−	−
*Ciconia ciconia (♀)*	−	−
*Ciconia ciconia (♂)*	−	−
Control *+ (♂)*	+	+
Control (NTC)	−	−

In addition, presence/absence experiments were performed on faecal samples. In both cases, the experimental runs were run in DA2 software (Design & Analysis Software Ver. 2.6.0; Thermo Fisher Scientific) under the criteria of positivity at Cq <38 threshold according to the estimates of efficacy and LOD.

### 2.8 Sex Ratio Analysis

The sex ratio (♀:♂) was assessed using the Chi‐square statistic test to confirm that its distribution followed Fisher's principle, which assumes a 1:1 sex ratio. The data were analysed as a whole or by sampling valleys as follows: Ambroz, Jerte and Tiétar (La Vera) valleys. *p* value was set to >0.05 to indicate no significant deviations from the 1:1 ratio.

## 3 Results

### 3.1 Sex Chromosome Target: Specificity, Efficiency and LOD Using Genomic DNA

Male and female gDNA were always subjected to amplification experiments for DBX and DBY in separate reactions but on the same plate. Both sex chromosome assays were shown to be specific in their ability to amplify DBY and DBX target genes using high‐quality desman gDNA, but the absence or presence (with enough DNA) of the Y chromosome in the female or male phenotypes, respectively, has been repeatedly corroborated as expected. Thus, female gDNA was consistently negative for the Y chromosome, the reason why it was excluded from further analysis. On the contrary, no amplification was observed with gDNA isolated from other mammalian sources, including moles and Mediterranean water shrews, as expected (Table 1). The latter species is particularly relevant as the Mediterranean water shrew shares the habitat of the desman in Extremadura. Additionally, NTCs (non‐template controls) were used for each gene and gDNA of known sex with negative results.

Regression analysis indicated that the linear range of both assays extends from 10^2^ to 10^0^, corresponding from 20 to 0.2 ng/µL DNA (Figure S1) with all *R* ^2^ >0.999. This allows us to state that linearity has been lost with gDNA from the fourth dilution onward.

Efficiency estimation was modest but valuable. Figure 1 shows the average efficiency (84%) using all datasets but ranged from 83% to 85% in three separate experiments. Overall, efficiencies were not significantly different between sexes or chromosomes (not shown), either globally, by assay or by chromosome. A general result was the loss of linearity at both gene targets in males and females from 10^−2^ dilution (0.1 total ng), which is why the sensitivity of the assay was performed from 10^−1^ dilution for the serial double dilution assay. Furthermore, the phenotypic female gDNA lost the DBX amplification signal from a 10^−2^ dilution and the male gDNA does so from the 10^−1^ dilution (Figure S1), which could suggest dose differences if a XX/XY sex‐determination system is assumed for the desman.

Probit analysis was used to determine LOD (DNA concentration at 99% detection probability [sensitivity%]). The LOD of both assayed chromosomes (DBX and DBY) was determined by preparing a twofold dilution from 0.2 ng/µL DNA, the last 10‐fold dilution within linearity. Each dilution series was prepared to have X and Y chromosomes within sex (two dilutions for male and one for female), and the experiment consisted of nine separate reactions of each one of the twofold decreasing DNA dilutions (Figure 1). Table 2 shows the positive replicate target calls for the different genera and chromosomes using the presence/absence detection module where they were automatically annotated (see Section 2), including the mean Cq values. The results indicated that the sensitivity at 99% probability was different for each chromosome and sex, due to the expected difference in X ploidy degree between sex as expected.

**TABLE 2:** Intra‐run variation for 10 twofold dilutions of DNA (Log_10_ [DNA]) from male chromosomes (Y and X) and X chromosomes from females using DBY and DBX assays. Dilution was prepared from the last 10‐fold dilution of the linear range of the assay. The Log_10_ concentration of DNA [DNA] and its corresponding averaged Cq at which Y/X chromosome on a male /X chromosomes on a female was simultaneously detected without RT‐qPCR failure is indicated in bold. The standard deviation (SD) and coefficient of variation percentage (CV%) are also highlighted.

	Chromosome: Y/X from male/X from female			
Log_10_ [DNA]	Mean Cq	positive	SD	CV%
0.000	31.65/28.36/27.72	9/9/9	0.34/0.22/0.01	1.26/0.79/0.04
−0.301	33.19/29.63/29.18	9/9/9	0.52/0.31/0.02	1.55/1.03/0.05
−0.602	33.13/30.95/30.38	9/9/9	1.8/0.23/0.32	5.44/0.76/1.07
−0.903	36.98/32.07/31.56	9/9/9	1.3/0.48/0.42	3.53/1.49/1.31
−**1.204**	**37.29/33.54/33.01**	**9/9/9**	**1.06/0.7/0.43**	**2.84/2.09/1.29**
−1.505	37.15/34.21/34.44	8/9/9	0.95/1.12/1.04	2.56/3.28/3.02
−1.806	38.9/35.11/35.26	6/8/9	1.51/0.77/0.69	3.87/2.2/1.97
−2.107	35.23/37.61/35.92	2/1/5	2.58/0/1.46	7.31/0/4.06
−2.408	39.7/37.34/36.39	3/3/3	1.13/0.67/0.63	2.86/1.81/1.72
−2.709	38.59/37.56/36.35	4/2/2	1.72/1.25/0.04	4.47/3.34/0.12

**FIGURE 2:**
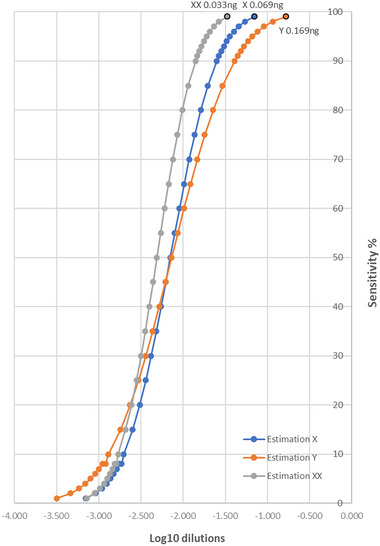
Probit analysis of twofold dilution series of positive amplicons by chromosomes within sex. The total estimated quantity of DNA at 99% sensitivity is indicated for Y and X in males and X in females.

The mean Cq results and the coefficient of variation observed for Y and X chromosomes in males and X chromosomes in females are summarized in Table 2. At least one replicate was lost after the fifth double dilution, especially for the Y chromosome. In addition, the standard deviation (SD) and percentage of the coefficient of variation (CV%) increased in a more random and stochastic manner after the fifth double dilution, also reaching maximum values for the differences between the different replicates. This fact pointed to the highest probability of technical failure after this dilution and signalling the threshold of a detectable amount of genetic material for both females and males. Accordingly, the Cq value was determined for each chromosome and sex (Table 2). Thus, the female and the male X chromosomes can be identified without doubt between the 34th and 35th cycle and 37th cycle for Y chromosomes, respectively. An additional result was obtained by estimation of the DNA concentration required for no technical failure of the assay based on LOD (Table 2). Y‐chromosome gDNA can be detected without failure with at least 0.166 total ng of gDNA, around the quantification cycle (Cq) of 37 as stated. Instead, X‐chromosome gDNA was successfully detected in males with 0.069 total ng (Cq 34–35), but in females, the estimated sensitivity was 0.033 total ng at a similar Cq. Consequently, twice as much gDNA was needed in males as in females, which was to be expected, as females carry a double dose of the X chromosome (Figure 2). Paradoxically, the Y chromosome required almost three times as much gDNA as the X chromosome using genetic material from the male. This result therefore quantified a dosage imbalance in the male genome in favour of the X chromosome, even though both chromosomes were expected to be equally represented for each target gene.

### 3.2 Sex Identification through Non‐Invasive Samples

The above results were especially dedicated to the desman conservation strategy through a non‐invasive sampling effort according to Ripa et al. (2024). Thus, a total of 178 faeces were successfully confirmed as belonging to *G. pyrenaicus* by RT‐PCR (Ripa et al. 2024). Subsequently, all of them were subjected to DBY and DBX diagnostics following the presence/absence procedure described here. All these samples were tested separately with the DBX and DBY assays, and a presence/absence analysis was performed adjusting 39 cycles as the cut‐off value in the presence/absence module in DA2. The positivity for DBY and DBX chromosomes was assessed one by one following the mean Cq threshold of each target obtained by LOD (Table 2). Thus, 49.9% of the samples rendered successful positive results, but from these a 49% showed only the X‐chromosome target and were therefore assigned to female specimens and 39% were from males with positive detection of both chromosomes (X and Y chromosomes). Furthermore, inconclusive or indeterminate results were obtained in 83 of the samples (Table 3). As a result, almost half of the faeces were successfully sexed overall, but with similar percentages in each of the valleys where desman had previously been found (Ripa et al. 2023, 2024), ranging from 47.8% to 53.3% (Table 3).

**TABLE 3:** Sex identification. Full sampling dates and locations with the different recognised populations. The *p*‐value and confidence interval analysis of the sex ratio is also shown.

Sex identification of *Galemys pyrenaicus* from the Central System Mountains in South‐western Spain						
	Total desman	Total sexing (%)	♀	♂	*χ* ^2^ *p* value	CI 95%
Whole sampling	178	88 (49.4)	49	39	0.450	0.440–0.460
Ambroz	67	32 (47.8)	17	15	0.860	0.853–0.867
Jerte	81	40 (49.4)	19	21	0.870	0.863–0.877
Vera (Tiétar)	30	16 (53.3)	12	4	0.074	0.069–0.079

For the whole sampling, no deviations from Fisher's principle of sex ratio (sex ratio 1:1) were observed, suggesting that the data obtained from male and female faeces were balanced (*p*‐value greater than 0.05). It was also corroborated for any of the population subdivisions following those described in the conservation plan of the desman published by the government of Extremadura (Ambroz, Jerte and La Vera, Table 3). The lowest amount of male faeces was found in La Vera, probably due to higher stochasticity as it coincides with a small sampling. Interestingly, these results were useful in the assessment of the presence of both sexes within sampling sites. Thus, the presence of female and male specimens, possibly female–male pairs, was also found at four, seven and two sampling riverside sections in Ambroz, Jerte and La Vera, respectively. Single females and single males were found in Ambroz and La Vera and in one site each in Ambroz and Jerte. It was therefore reasonable to assume the presence of breeding pairs at each of these thirteen sites.

## 4 Discussion

Historically, sex determination in the desman based on external genitals has been controversial, from Peyre (1957a, 1957b) to the recent phenotypic studies of González‐Esteban et al. (2003). Subsequently, with the advent of molecular biology, Vidal et al. (2010) described the first sex determination protocol in the Iberian desman, relying on end‐point PCR techniques to efficiently sex the gDNA of this species. However, these authors found very low success (10%) when faeces were the source of genetic material. PCR‐based techniques, especially the RT‐qPCR variant assays, have recently been used for non‐invasive or eDNA monitoring of *G. pyrenaicus* (Ripa et al. 2023, 2024, Forthcoming) and other species (Abe et al. 2001; O'Neill et al. 2013; Matejusová et al. 2013). The present study describes the utility of the RT‐qPCR assay for the detection of both sex chromosomes (X and Y chromosomes) of *G. pyrenaicus* using both gDNA and faecal DNA, with increased sensitivity using hydrolysis probes for both X and Y chromosomes. The DBX and DBY assays showed moderate efficiency but good analytical sensitivity and high specificity, as no cross‐reactivity was observed between different chromosomes within the desman or when testing DNA from other mammals. In this study, the success rate was 0.5 with an eDNA template from faeces, based on a previous assignment to desman species (Ripa et al. 2024). It is likely that success could be increased by replicating to exhaustion of the extracted DNA.

The DNA extracted from the faeces sampled was often scarce and, as in our case, had to be used for many other tasks and studies with similar conservation relevance, such as the study of species health (Ripa et al. 2023) or mitochondrial sequencing (Ripa et al. 2024 and references therein). Furthermore, as noted by Vidal et al. (2010), the quality of faecal DNA varies greatly depending on the environmental conditions to which each sample was exposed. In this regard, a potential improvement of RT‐qPCR for species identification or sex determination in faecal samples has been demonstrated in several studies, as problems of DNA degradation and fragmentation could be overcome by using smaller target sizes (Morin et al. 2005; Moran et al. 2008; O'Reilly et al. 2008; Matejusová et al. 2013). However, not many studies give equal importance to the assessment of analytical exclusivity and inclusivity or also detection limits in their studies. Like us, O'Neill et al. (2013) also considered rejecting samples that did not detect the target species, as negative results are sometimes expected in many environmental surveys.

In addition to species verification (Ripa et al. 2023, 2024), sexing of individuals is considered crucial in ecological studies, especially those of elusive or critically endangered species. As a result, this technique is increasingly used not only for sexing but also to provide information on genetic structure, size, genetic diversity and relatedness within a population (Waits and Paetkau 2005; Arandjelovic et al. 2010; Oliveira et al. 2010; Ruiz‐González et al. 2012; Ripa et al. 2024).

The detection performance of the test was determined by three separate experiments. In two of these, the efficiency of the test was evaluated for the detection of the X chromosome in females and males, respectively, and in a third for the Y chromosome in males using gDNA and six decimal dilution series. In all three experiments, the efficiencies were modest (∼85%) when compared with the percentage efficiencies of O'Neill et al. (2013) in otters, who obtained individual efficiencies of ∼93%–95% for the X and Y chromosomes, respectively. However, in this study, a regression coefficient greater than 99.9% was obtained, indicating a good correlation between DNA concentration and Cq. These results agree with those obtained by O'Neill et al. (2013) or Bhoora et al. (2018), suggesting the precise nature of this methodology, coupled with sufficient specificity for sexing with DBX and DBY genes.

An important difference compared to other studies was that the LOD of the assay was also studied. This parameter is an important aspect to be investigated when working with a limited amount of DNA, such as those from faeces, as it underlies the sensitivity of the test. According to the LOD results, it took twice as much genetic material from a male as from a female (0.069/0.033 ∼ 2; 99% LOD) to detect the DBX gene target with the same probability (Figure 2). This result was very suggestive, as it is in line with what is expected for an XX/XY sex‐determination system in the desman, the most common sex determination system in mammals. Consistently, the Y chromosome was completely absent from female gDNA, as confirmed by Vidal et al. (2010). However, the DBY assay required ∼2.5 times more male gDNA than the DBX assay to detect the Y and X chromosomes, respectively, suggesting at least the multicopy nature of the DBX gene due to no differences in efficiency between the two assays. The heterogametic sex (males) showed less amplifiable genetic material than females from sex chromosomes, suggesting new challenges as to whether these DBX and DBY genes are located outside the pseudoautosomal regions present on mammalian sex chromosomes. This raised important questions, as many genes in the pseudoautosomal regions are also essential for normal development (Helena Mangs and Morris 2007).

Although more research needs to be done to unravel the issue of multicopy for DEAD‐Box genes, it is proving to be an interesting research topic as follows. While mammalian sex is genetically determined by differentiated testicular or ovarian tissue, resulting in sex‐specific anatomical, hormonal and behavioural differences (Capel 2017), a striking exception to this paradigm occurs in moles (family *Talpidae*). In at least eight species of moles, XX‐genotypic females have intersex phenotype (Barrionuevo et al. 2004), justifying the development of ovotestes instead of single ovaries (Real et al. 2020), as in the desmans. Indeed, females of these species show high androgen synthesis, masculinized external genitalia (Jiménez, Burgos, and Barrionuevo et al. 2023), as well as prominent muscles and aggressive behaviour (Haeck 1969), traits that probably represent adaptations to their particular lifestyle. A tandem triplication at the CYP17A1 locus (encoding a key enzyme controlling androgen synthesis, Hanukoglu 1992) has been associated with increased androgen production and strength in moles (*T. occidentalys*), suggesting a role in the masculinization of female moles. Thus, in contrast to the general pattern in mammals where males show higher levels than females, this higher gene dosage, due to duplication and functional changes in regulatory sequences, may explain similar levels of circulating androgens in females as in males of some talpids, implying phenotypic adaptations (Real et al. 2020).

The DBX genes belong to a large family of DEAD‐box genes that are expressed in oocytes of both mammalian and non‐mammalian species. Such genes showed differential expression throughout oogenesis and in mature oocytes, suggesting a role in RNA production, processing and transport during oogenesis (Longo et al. 1996). Expression studies of the homologous DBX gene in the female germline of drosophila, frogs, zebrafish, mice and humans strongly suggest that this function is conserved and most likely plays a functional role in folliculogenesis. Impairment of female fertility has been linked to deletion or mutation of genes that disrupt the female germline DBX function (Fassnacht et al. 2006). Consequently, just as tandem triplication is associated with increased circulating androgens in males, we wondered about the role of the apparent finding of the multicopy nature of the DBX gene and also about the effect of increased gene dosage of these genes in both sexes on desman. We also wondered whether the largest copy of DBX might compensate for the higher androgen expression found in intersex females.

The performance of these assays has been further studied with field samples collected throughout the range of the desman in Extremadura (Central System Mountains), which includes the three main populations (Ripa et al. 2023, 2024). At first sight, DNA‐based sexing facilitates establishing the sex ratio of males and females despite adult males and females having different detectability due to sex differences in behaviour and ecology, and adults may form sex‐specific aggregations during breeding and/or nonbreeding periods. Furthermore, non‐invasive results supported Fisher's principle as would be expected for a XX/XY sexual system. Overall, our analyses showed no evidence of sex ratio bias, which may reflect the lack of sex differences in maturation, dispersal and survival between populations or globally (Székely et al. 2014). However, there are apparently singleton males or females at some sampling sites (∼30% sites), suggesting a sex‐balanced dispersal in this species. Under scenarios of male‐biased sex ratios in species characterized by ‘conventional’ sex roles, males tend to be more aggressive and fight more often to directly outcompete rivals (Weir et al. 2011). Similarly, a female‐biased sex ratio has been associated with increased aggression in females (Cheney et al. 2012), as expected in some species of the family Talpidae because females showed masculinization as well as prominent muscles and aggressive behaviour (Haeck et al. 1969). This fact may support similar dispersal behaviour of males and females in species with ovotest such as moles and desmans, which has been shown to be compatible with diploid sex determination (Chau et al. 2018).

## 5 Conclusions

This study demonstrated the successful application of RT‐qPCR assays for molecular sex determination using genetic material from faeces and tissues of the Iberian desman (*G. pyrenaicus*). The specificity and sensitivity of both assays were also tested for the first time. The reliability of the test for the desman was confirmed both by obtaining a uniform sex ratio distribution even with non‐invasive samples and its value in molecular ecology studies for the assignment of singles or breeding pairs of these endangered species. In addition, new insights into the relevance of genes with copy number variation, especially those linked to the sex chromosomes, such as the DEAD‐Box in the desman, should be highlighted.

## Ethics Statement

The authors have nothing to report.

## Conflicts of Interest

The authors declare no conflicts of interest.

## Supporting information



Figure S1 Efficiency as a function of dilution, sex and gene of interest using six serial dilutions.
Table S1 Used sequence adjusted to RT‐Product size for Primer and Probes design from Vidal et al. (2010)

## Data Availability

The data presented in this study are available upon request from the author.
