# Supplementary material for: Molecular Method Based on Hydrolysis Probe Assays to Identify the Sex Chromosomes of Iberian Desman (Galemys pyrenaicus) Using Non‐Invasive Sampling
Source: Integr Zool. 2024 Dec 8;20(5):1047–55. doi: 10.1111/1749-4877.12933 (PMC12463749; doi:10.1111/1749-4877.12933)
Supplement: Supplementary file 1 — Figure S1 Efficiency as a function of dilution, sex and gene of interest using six serial dilutions. Table S1 Used sequence adjusted to RT‐Product size for Primer and Probes design from Vidal et al. (2010) [file INZ2-20-1047-s001.doc]

**Figure S1**
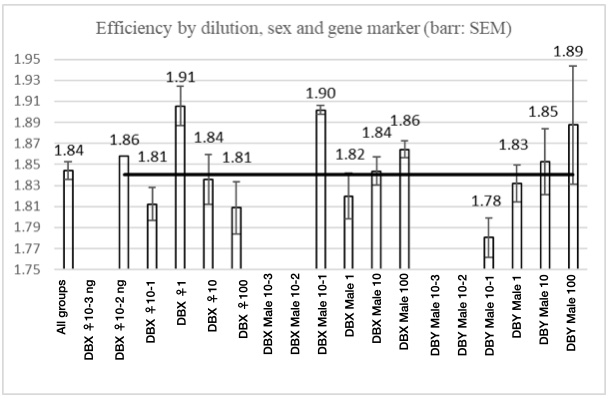
Efficiency as a function of dilution, sex and gene of interest using six serial dilutions.

**Table S1** Used sequence adjusted to RT-Product size for Primer and Probes design from Vidal et al. (2010)

| **Galemys pyrenaicus DBX gene, partial sequence** | |
| --- | --- |
| NCBI LOCUS DNA Accession Number: FJ638891 Total sequence size: 1477 bp | |
| Source | /organism="*Galemys pyrenaicus*" |
|  | /mol_type="genomic DNA" |
|  | /db_xref="taxon:[202257](https://www.ncbi.nlm.nih.gov/Taxonomy/Browser/wwwtax.cgi?id=202257)" |
|  | /chromosome="X" |
|  | /gene="DBX" |
|  | /note="coding region not determined" |
|  | /Selected Start to End oligonucleotide design segment = “AACTGCAGCTTTTCTCTTGCCCATTTTGAGTCAGATTTATTCAGATGGTCCAGGAGAGGCTCTGAGGGCCATGAAGGTAAATATTTCTTTACAAAATGGGAAATTATTGTTGACGGCTTT” |
|  | /Used segment size Chromosome X =120 bp |
| **Galemys pyrenaicus DBY gene, partial sequence** | |
| NCBI LOCUS DNA Accession Number: FJ638890 Total sequence size: 1415 bp | |
| source | /organism="*Galemys pyrenaicus*" |
|  | /mol_type="genomic DNA" |
|  | /db_xref="taxon:[202257](https://www.ncbi.nlm.nih.gov/Taxonomy/Browser/wwwtax.cgi?id=202257)" |
|  | /chromosome="Y" |
|  | /gene="DBY" |
|  | /note="coding region not determined" |
|  | /Selected Start to End oligonucleotide design segment =“AGTCTGTAGTGGGGATTGTATTGAGACATAACCCCGGGTCCACTGGGGGACCCTTGATAAACTTTTGCTTTAAAGTTTTATAAGTACTTAGTGAGACTTTTATAAATAATGCCAAATATATTTCATTTTTGTTTCTTGCCAGCTTACT” |
|  | /Used segment size Chromosome Y= 148 bp |
